# High prevalence of TB multimorbidity among adults of a tertiary hospital in Sierra Leone: a cross-sectional study

**DOI:** 10.1186/s13104-023-06476-y

**Published:** 2023-11-16

**Authors:** Sulaiman Lakoh, Patricia Lombeh Vamboi, Abdoul Risgou Ouédraogo, Olukemi Adekanmbi, Gibrilla F. Deen, James B. W. Russell, Ahmed Sankoh-Hughes, Joseph B. Kamara, Joseph Edwin Kanu, George A. Yendewa, Emmanuel Firima, André F. S. Amaral

**Affiliations:** 1 College of Medicine and Allied Health Sciences, University of Sierra Leone, Freetown, Sierra Leone; 2 Government of Sierra Leone, Ministry of Health and Sanitation, Freetown, Sierra Leone; 3 Sustainable Health Systems Sierra Leone, Freetown, Sierra Leone; 4 Division of Pulmonology, Training and Research Unit in Health Sciences, University Joseph KI-ZEBRO, Ouagadougou, Burkina Faso; 5 Department of Medicine, College of Medicine, University of Ibadan, Ibadan, Nigeria; 6 Department of Medicine, University College Hospital, Ibadan, Nigeria; 7 Department of Medicine, Case Western Reserve University School of Medicine, Cleveland, OH USA; 8 Division of Infectious Diseases and HIV Medicine, University Hospitals Cleveland Medical Center, Cleveland, OH USA; 9 Johns Hopkins Bloomberg School of Public Health, Baltimore, MD USA; 10 Clinical Research Unit, Department of Medicine, Swiss Tropical and Public Health Institute, Basel, Switzerland; 11 University of Basel, Basel, Switzerland; 12 SolidarMed, Maseru, Lesotho; 13 Centre for Multidisciplinary Research and Innovation, Abuja, Nigeria; 14 National Heart and Lung Institute, Imperial College London, London, UK

**Keywords:** TB multimorbidity, Diabetes, Hypertension, Tuberculosis, Obesity, HIV

## Abstract

**Objective:**

Tuberculosis (TB) is a leading cause of death globally, with approximately 1.5 million deaths in 2020. TB often coexists with chronic communicable and non-communicable diseases, but data to determine the extent of comorbid diseases are limited. In this study, we aimed to assess the prevalence of TB multimorbidity and its risk factors in a tertiary hospital in Sierra Leone. This is a cross-sectional study of 240 adults with microbiologically-confirmed TB at Connaught Hospital in Freetown, between March and May 2022. Logistic regression analysis was used to identify factors associated with TB multimorbidity.

**Results:**

The mean age of the patients was 37 years. More than two-thirds were males and about the same number had two or more chronic diseases. The most common were hypertension (47.9%) and diabetes (24.2%). Patients under 35 years of age were less likely to have TB multimorbidity (< 25 years: adjusted OR 0.07, 95%CI 0.01–0.6; 25–34 years: adjusted OR 0.2, 95%CI 0.01–0.9). We report a high prevalence of comorbid diseases among TB patients in the largest treatment center in Sierra Leone, with hypertension and diabetes being the most common. These findings support the current call for addressing comorbid non-communicable diseases in TB patients through integrated care.

## Introduction

Multimorbidity, defined as the co-existence of two or more chronic communicable and/or non-communicable diseases, is a growing global health problem [1]. In high-income countries, about 30% of adults have experienced multimorbidity at some point [2]. Although the exact burden of multimorbidity in low- and middle-income countries is unknown, prevalence in these countries is rising due to epidemiological transition caused by lifestyle changes, economic improvements, and changing environmental factors [3].

Tuberculosis (TB) is a leading infectious cause of death globally, with approximately 1.5 million TB deaths reported in 2020 [4]. TB often coexists with chronic communicable and non-communicable diseases, thus increasing management complexity and adversely affecting health and socioeconomic outcomes [5]. Despite these challenges with TB care, there are limited data on the prevalence of multimorbidity among TB patients in most countries of sub-Saharan Africa. A small number of observational studies have only considered individual chronic diseases in TB patients [6–8]. In a recent systematic review of TB multimorbidity, the prevalence of diabetes and HIV among TB patients in the African region was as high as 10.4% and 42.3%, respectively [9].

Sierra Leone, a low-income country in West Africa, has one of the highest TB burdens in the world [4, 10]. Superimposed on these challenges of chronic communicable disease is the high burden of hypertension (22%) and diabetes (10%) reported in the general population of Sierra Leone, which reinforces the need to know more about multimorbidity among TB patients [11, 12].

To date, however, there is no structure in the National TB Control Program to assess and manage multimorbidity in patients with TB, except HIV. In this study, we aimed to assess the prevalence of TB multimorbidity (hypertension, diabetes, obesity and HIV) and its risk factors among adult TB patients attending an urban tertiary hospital in Sierra Leone.

## Materials and methods

### Study design and setting

We used a cross-sectional design to collect primary data on TB multimorbidity in Connaught Hospital, which is Sierra Leone's national referral hospital with a capacity of 300 beds. The hospital is affiliated with the College of Medicine and Allied Health Sciences of the University of Sierra Leone [13]. Connaught Hospital's Chest Clinic provides outpatient and inpatient TB diagnosis and treatment services, including a Directly Observed Short-Term Treatment (DOTS) program. Owing to its location in the country's main referral hospital, the Chest Clinic provides services to the largest number of TB patients in Sierra Leone [14].

### Study population, sampling and data collection

Between March 2022 and May 2022, we recruited non-randomly 240 participants at the Chest Clinic. Two research assistants, trained on the measurement of blood pressure and anthropometric parameters and phlebotomy, collected the data. We used a paper-based questionnaire to collect socio-demographic, clinical, anthropometric, and laboratory information.

**Fig. 1:**
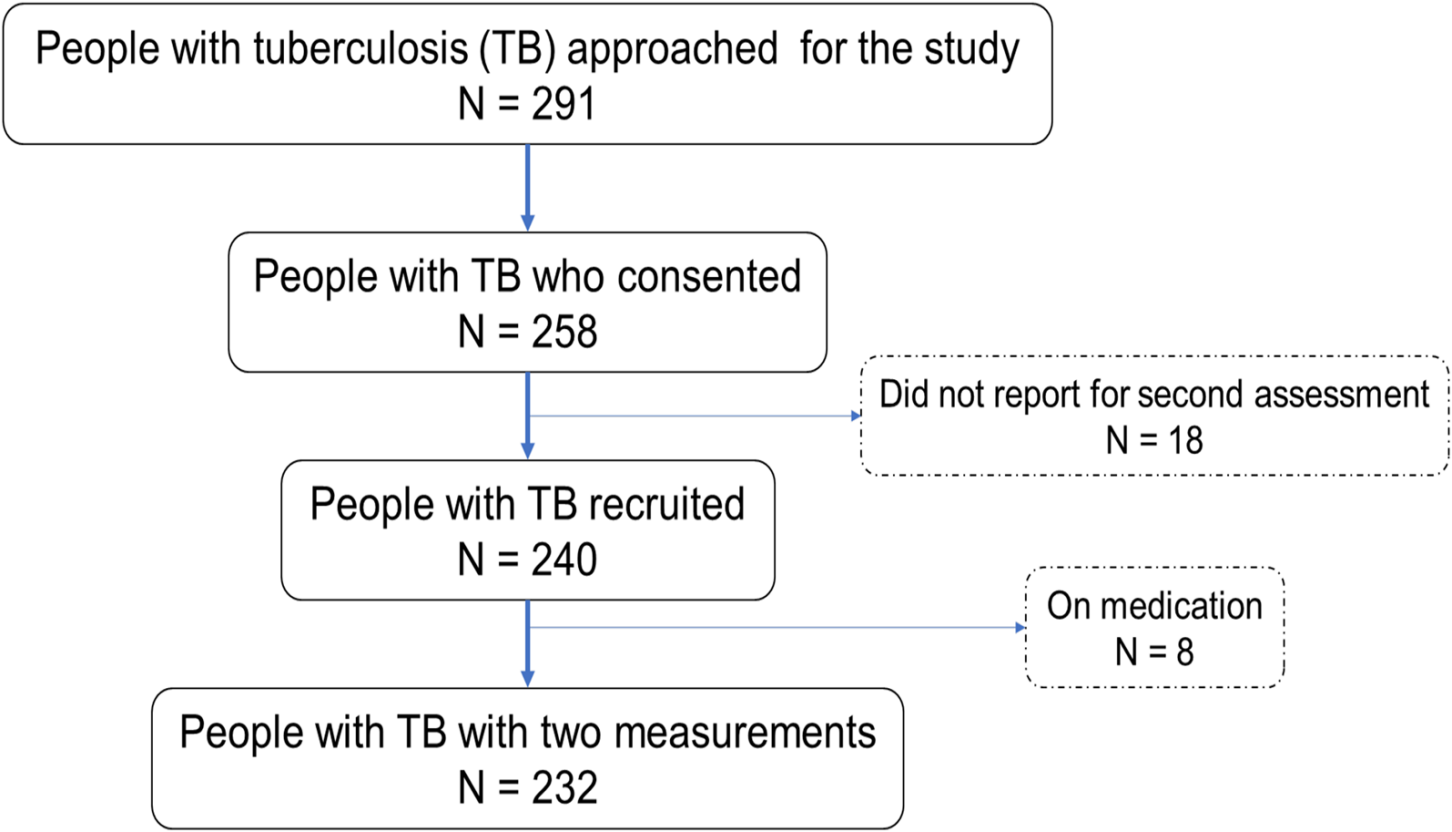
Recruitment details of people with tuberculosis for the study of multimorbidity

All microbiologically confirmed TB patients aged 18 years or older, including those newly diagnosed or receiving anti-TB treatment, were eligible, regardless of the treatment duration. Patients with extrapulmonary TB, and those who refused to consent to a second plasma glucose or blood pressure measurements were excluded from the study. Figure 1 shows the recruitment details of people with TB for the assessment of multimorbidity.

### Assessment of blood pressure and fasting plasma glucose

We measured the blood pressure and fasting plasma glucose twice, at baseline and at least three days thereafter except where the result was unequivocally high or the patient was on medication.

We measured the blood pressure using a well-calibrated oscillometric manual device as recommended by the American College of Cardiology/American Heart Association (ACC/AHA) guidelines [15]. The average of the two blood pressure measurements was used to categorize patients as follows: (1) normal: < 120/80 mmHg; (2) elevated: systolic blood pressure (SBP) > 120–129 mmHg and diastolic blood pressure (DBP) < 80 mmHg; (3) stage 1 hypertension: SBP > 130–139 mmHg or DBP ≥ 80–89; (2) stage 2 hypertension: SBP ≥ 140 mmHg or DBP ≥ 90 mmHg. We also considered patients to have hypertension if they were on antihypertensive medication, regardless of their average blood pressure records.

We screened for impaired glucose tolerance by measuring the fasting plasma glucose in accordance with the American Diabetes Association (ADA)’s criteria [16]. The average fasting plasma glucose after two measurements separated at least three days apart was used to classify fasting plasma glucose levels as follows: (1) prediabetes, defined as fasting plasma glucose level of 5.6–6.9 mmol/L (2) diabetes, defined as fasting plasma glucose level ≥ 7.0 mmol/L, or being on antidiabetic medications, regardless of the fasting plasma glucose.

### Assessment of the body mass index and waist circumference

The body mass index (BMI) was defined as a person's weight in kilograms divided by their height in meters square and categorized accordingly into underweight (< 18 kg/m^2^), normal weight (18–25 kg/m^2^), overweight (25–30 kg/m^2^) and obesity (≥ 30 kg/m^2^) [17].

We used a tape measure to assess each patient's waist circumference. Abdominal obesity was defined as a waist circumference greater than 94 cm in men and 80 cm in women using a recent European consensus statement [18].

### TB multimorbidity

We defined TB multimorbidity as the co-existence of TB with one or more of HIV, obesity, hypertension and diabetes mellitus.

### Data analysis

Data analysis was performed using SPSS Version 28.0 (IBM Corp; Armonk, NY, USA). Categorical variables were reported as frequencies and percentages.

A logistic regression model was used to identify risk factors associated with TB multimorbidity. Variables that attained a *p*-value < 0.2 in the univariable analysis were included in the multivariable regression model. Associations were reported as crude (OR) and adjusted odds ratios (aOR) with 95% confidence intervals (CI), with statistical significance set at *p* < 0.05.

## Results

### Socio-demographic details

The mean age of the 240 patients enrolled in this study was 37 (SD 14) years, with a range of 18 to 83 years. Of these patients, 170 (70.8%) were males (sex ratio = 2.4), 116 (48.3%) were single, 174 (72.5%) worked in the informal sector, and 132 (55%) had secondary education. Cigarette smoking was reported by 51 (21.2%) patients (Table 1).

**Table 1:** Socio-demographic characteristics of the participants (N = 240)

Socio-demographic variables	Frequency	Percentage
Age (yr)		
< 25	40	16.7
25–34	83	34.6
35–44	52	21.7
45–54	34	14.2
≥ 55	31	12.9
Sex		
Female	70	29.2
Male	170	70.8
Marital status		
Single	116	48.3
Married	105	43.8
Separated/widowed/divorce	19	7.9
Occupation		
Unemployed	16	6.7
Student	30	12.5
Informal sector	174	72.5
Formal sector	10	4.2
Retired	10	4.2
Level of education		
None	37	15.4
Primary	24	10.0
Secondary	132	55.0
Tertiary	47	19.6
Smoking and substance use		
Cigarette smoking	51	21.4
Alcohol use	77	32.1
Family history		
Diabetes	25	10.4
Hypertension	36	15.0

### TB and comorbidity anthropometric, blood pressure and fasting glucose measurements

TB was diagnosed using Xpert MTB/Rif in 185 (77.1%) cases. Most (92.1%) cases were new TB diagnosis and many (69.6%) were in the intensive phase of TB therapy. Few (1.3%) patients were overweight, but a substantial proportion (11.7%) had truncal obesity (Waist circumference > 94 cm for men or > 80 cm for women) (Table 2).

**Table 2:** TB details, anthropometry, plasma glucose and blood pressure (N = 240)

Variable	Frequency	Percentage
Mode of TB diagnosis		
Xpert MTB/Rif	185	77.1
AFB smear	53	22.1
Urinary TB LAM	2	0.8
Types of patients		
New	221	92.1
Relapse	14	5.8
Treatment failure	3	1.3
Treatment interruption	2	0.8
Phase of TB therapy		
Intensive phase	167	69.6
Continuation phase	73	30.4
Waist circumference (cm)		
Men > 94 or women > 80	28	11.7
Men ≤ 94 οr women ≤ 80	212	88.3
BMI		
Underweight	202	84.2
Normal weight	34	14.2
Overweight/obesity	4	1.7
Fasting plasma glucose (mmol/l)		
Normal (≤ 5.5)	58	24.2
Prediabetes (5.6–6.9)	124	51.7
Diabetes (≥ 7 or using medication)	58	24.2
Blood pressure (mmHg)		
Normal	91	37.9
Elevated	31	12.9
Stage 1 hypertension	98	40.8
Stage 2 hypertension	20	8.3
HIV status		
Positive	49	20.6
Negative	191	79.4

TB-LAM: Tuberculosis lipoarabinomannan BMI: Body mass index MTB: *Mycobacterium tuberculosi*s HIV: Human immunodeficiency virus

**Fig. 2:**
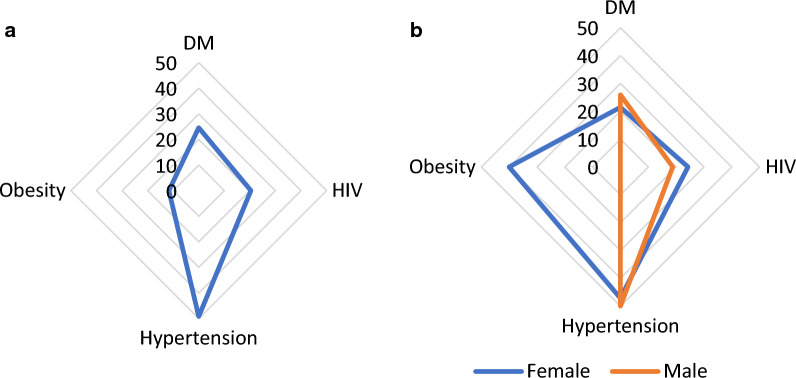
Distribution of TB multimorbidity in percentage **a** in the total sample and **b** by sex

About 70.8% (95% CI: 63.8 to 75.7) of patients with TB had multimorbidity. Amongst TB patients with multimorbidity, the prevalence of comorbid illness was as follows: hypertension 49.1% (95% CI; 41.4 to 54.4), diabetes mellitus 24.2% (95% CI:18.9 to 30.1), HIV 20.4% (95% CI: 15.5 to 26.1) and truncal obesity 11.7% (95% CI: 7.9 to 16.4)). Three patients (1.3%) had all four assessed co-morbidities (Fig. 2).

### Factors associated with TB multimorbidity

In univariable analysis, young adults (< 25 years: OR 0.03, 95% CI 0.0–0.2; 24–34 years: OR 0.06, 95% CI 0.0–0.5; 35–44 years: OR 0.1, 95% CI 0.0–0.9), those with primary education (OR 0.27, 95% CI 0.1–0.9), or still studying (OR 0.04, 95% CI 0.002–0.78) were less likely to show TB multimorbidity. Patients who were not single (married: OR 2.3, 95% CI 1.3–4.1; divorced/separated/widowed: OR 5.4, 95% CI 1.2–24.4) or had family history of hypertension (OR 2.9, 95% CI 1.1–7.8) were more likely to have TB multimorbidity.

After adjusting for confounders, only age was significantly associated with TB multimorbidity, with patients under the age of 35 years (< 25 years: aOR 0.07, 95% CI 0.01–0.6; 25–34 years: aOR 0.2, 95% CI 0.01–0.9) being less likely to have two or more diseases (Table 3).

**Table 3:** Bi- and multivariable analysis of TB multimorbidity and variables of interest

Variables	TB Multimorbidity		Crude odds ratio (95% Confidence interval)	P value	Adjusted odds ratio (95% Confidence interval)	P value
	Yes N (%) 170(70.8)	No N (%) 70 (29.2)				
Sex						
Female	54(31.8)	16(22.9)	1.6 (0.8–2.9)	0.170	1.9(0.9–4.0)	0.116
Male	116(68.2)	54(77.1)	1		1	
Age						
< 25	18(10.6)	22(31.4)	0.03(0.0–0.2)	0.001	0.07 (0.01–0.6)	0.019
25–34	53(31.2)	30(42.9)	0.06(0.0–0.5)	0.007	0.2(0.01–0.9)	0.047
35–44	40(23.5)	12(17.1)	0.1(0.0–0.9)	0.04	0.2(0.02–1.7)	0.1
45–54	29(17.1)	5(7.1)	0.2(0.0–1.8)	0.1	0.3(0.03–3.3)	0.4
≥ 55*	30(17.6)	1(1.4)	1		1	
Marital status						
Single	71(41.8)	45(64.3)	1		1	
Married	82(48.2)	23(32.9)	2.3(1.3–4.1)	0.007	0.9(0.5–1.9)	0.9
Divorced/separated/widowed	17(10)	2(2.9)	5.4(1.2–24.4)	0.029	1.6(0.3–8.8)	0.6
Education						
None	31(18.2)	6(8.6)	1		1	
Primary	14(8.2)	10(14.3)	0.3(0.1–1.1)	0.03	0.4(0.1–1.3)	0.1
Secondary	88(51.8)	44(62.9)	0.4(0.2–1.1)	0.05	0.7(0.2–1.8)	0.4
Tertiary	37(21.8)	10(14.3)	0.7(0.2–2.2)	0.6	1.4(0.4–5.8)	0.6
Occupation						
Unemployed	12(7.1)	4(5.7)	0.1(0.01–2.8)	0.2	1.1(0.1–10.3)	0.9
Student	14(8.2)	16(22.9)	0.04(0.002–0.8)	0.03	0.5(0.06–3.3)	0.4
Informal sector	126(74.1)	48(68.6)	0.1(0.01–2.2)	0.2	1.2(0.2–7.3)	0.9
Formal sector	8(4.7)	2(2.9)	0.2(0.01–3.9)	0.3	0.9(0.0–33.4)	0.9
Retired	10(5.9)	0	1		1	
Alcohol consumption						
No	113(66.5)	50(71.4)	1			
Yes	57(33.5)	20(28.6)	1.3(0.7–2.3)	0.5		
Cigarette smoking						
No	130(77.4)	57(81.4)	1			
Yes	38(22.6)	13(18.6)	1.3(0.6–2.6)	0.489		
Types of patients						
New	156(91.8)	65(92.9)	1			
Relapse/treatment failure/treatment interruption	14(8.2)	5(7.1)	1.2(0.4–3.4)	0.776		
Family history of Hypertension						
No	139(81.8)	65(92.9)	1		1	
Yes	31(18.2)	5(7.1)	2.9 (1.1–7.8)	0.035	2.37(0.81–6.92)	0.114
Family history of Diabetes						
No	152(89.4)	63(90)	1			
Yes	18(10.6)	7(10)	1.06 (0.4–2.7)	0.892		

## Discussion

This study is the first to examine multimorbidity in adult patients with TB cared for at a national referral hospital in Sierra Leone. Our study showed that 70.8% of TB patients in this hospital had one or more additional chronic diseases.

A number of studies have provided data on multimorbidity in Africa, but none has focused specifically on TB multimorbidity. In South Africa, a multimorbidity prevalence of 22% was reported in a peri-urban healthcare setting, although not exclusive for TB patients [18]. An earlier study reported a lower TB multimorbidity prevalence of 1.14% in Brazil, which may reflect a lower national prevalence of HIV or other comorbidities [19].

Among the comorbid diseases reported in the TB population, hypertension was the most common, with a reported prevalence of 49%, which was higher than the 22% prevalence in the general population of Sierra Leone. The ACC/AHA guidelines, which we used in this study, have a lower blood pressure threshold of > 80 mmHg for diastolic blood pressure, compared to the threshold of ≥ 90 mmHg defined in other guidelines [15, 20]. Thus, the difference in prevalence between the two studies could be explained by differences in diastolic blood pressure measurement thresholds, or may represent a true reflection of the hypertension burden in this population. Nonetheless, evidence from meta-analyses of observational studies suggests that elevated blood pressure and stages 1 or 2 hypertension, as defined in this study, are associated with increased cardiovascular-related mortality if left untreated [21]. Therefore, in settings of poor health-seeking behaviors, healthcare professionals should employ practical approach to detecting and managing blood pressures at lower thresholds to prevent cardiovascular disease risk, end-stage renal disease, and death.

Similar to hypertension, the prevalence of comorbid diabetes in TB patients in this study was higher than that reported in the general population of Sierra Leone [11]. The World Health Organization proposes a collaborative framework to integrate diabetes care into TB prevention and control services, as the two conditions can negatively impact each other. The recommendations were provisional as the evidence from which it was based was weak [22]. Thus, this study will add to the body of evidence on the need for the integration of diabetes care to TB services.

Despite the low national HIV seroprevalence of 1.7% [23], previous studies have reported a higher HIV burden among TB patients in the national referral hospital of Sierra Leone [14, 24]. Because of this and the fact that the hospital has concentrated HIV cases, it is not surprising that a high prevalence of HIV among tuberculosis patients is reported in this study [25].

Although abdominal obesity was the least comorbid condition among TB patients in this study, its high prevalence in this population is unexpected because TB patients are most often underweight. Nonetheless, this finding must be reported with caution due to the use of European thresholds to define obesity in this study [16].

Among patients with TB, young adults were less likely to have multimorbidity. The association between chronic comorbid disease and age is well established in the literature [25]. Previous studies reporting high prevalence of comorbidities in older populations support our finding that young adults under 35 are less likely to develop TB multimorbidity [19, 26]. In contrast to our study, a previous study reported a high incidence of TB among Brazilian women [19].

Our study has strengths and limitations. Data were collected sequentially from a diverse population in the largest TB treatment center in the country, and blood pressure and anthropometry were measured by trained personnel in accordance with international standards. However, as a single-center study conducted at a national referral hospital, the findings cannot be generalized to the general TB population. Nonetheless, these findings can be strengthened and used to advocate for the integration of chronic communicable and noncommunicable diseases in TB prevention and control.

## Conclusion

In conclusion, we report a high prevalence of comorbid diseases among TB patients in the largest treatment center in Sierra Leone, with hypertension and diabetes being the most common. These findings support the current call for addressing comorbid non-communicable diseases in TB patients through integrated care in low-income countries, where the prevalence of non-infectious diseases is increasing.

## Data Availability

The data is available at the University of Sierra Leone repository and will be available upon request.

## Abbreviations

ACC: American College of Cardiology
ADA: American Diabetes Association
AHA: American Heart Association
BMI: Body mass index
BP: Blood pressure
HIV: Human immunodeficiency virus
MTB: *Mycobacterium tuberculosis*
NCD: Non-communicable diseases
TB: Tuberculosis
WHO: World Health Organization
